# Inferior epigastric artery pseudoaneurysm following abdominal paracentesis in a patient with chronic kidney failure: A case report and review of literature

**DOI:** 10.1002/ccr3.5535

**Published:** 2022-03-10

**Authors:** Behnam Kian, Arash Teimouri

**Affiliations:** ^1^ 48435 Medical Imaging Research Center Shiraz University of Medical Sciences Shiraz Iran

**Keywords:** complication, imaging, inferior epigastric artery, paracentesis, pseudoaneurysm

## Abstract

Inferior epigastric artery (IEA) pseudoaneurysm is a rare complication following abdominal wall procedures near the artery. This is a case of inferior epigastric artery pseudoaneurysm after therapeutic paracentesis for large‐volume ascites caused by chronic kidney failure. The patient was operated on, and the artery was ligated.

## Graphical abstract

A paracentesis procedure could cause complications if the physician ignores some simple rules. IEA pseudoaneurysm is a rare complication of the procedure, so diagnosing this condition is crucial to treat early and avoid rupture.

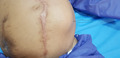

## 1 INTRODUCTION

Pseudoaneurysm is one of the complications after injury to the arterial wall, which involves one or two layers, unlike a true aneurysm, which affects all three layers. Pseudoaneurysm is a pulsatile mass that connects to the artery after disruption of its wall.^1^

The inferior epigastric artery (IEA) is one branch of the external iliac artery that supplies the anterior abdominal wall. IEA pierce the transversalis fascia on each side of the rectus sheath and enter the rectus abdominis muscle. We reported an IEA pseudoaneurysm after paracentesis, a rare complication after abdominal wall intervention.^2^

Indications for paracentesis in a patient with chronic kidney failure are varied and include the following: rule out the spontaneous bacterial peritonitis, finding etiology of new ascites, and therapeutic management of abdominal discomfort and respiratory distress.^3^

## 2 CASE PRESENTATION

**FIGURE 1:**
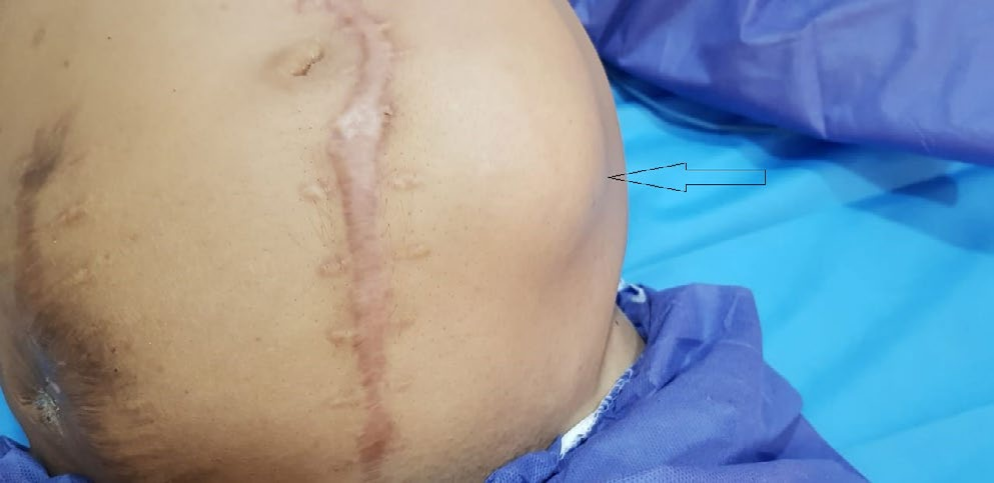
A 53‐year‐old man presented to the emergency department with a 2‐day history of left lower quadrant palpable mass (arrow)

A 53‐year‐old man presented to the emergency department with a 2‐day history of left lower quadrant palpable mass (Figure 1). Two days before presentation, abdominal paracentesis because of large ascites fluid, which caused abdominal discomfort, was performed for the patient, and swelling began after that. The patient started dialysis in 2016. Past medical history included renal failure, hypertension, and smoking.

On admission, his vital signs were a temperature of 37°C, heart rate of 86 bpm, blood pressure of 150/85, and respiratory rate of 20 per minute.

Physical examination revealed a non‐tender and non‐compressible mass in the left side of the umbilicus. Abdominal distention was noticed; nonetheless, no trill or bruit was detected.

**FIGURE 2:**
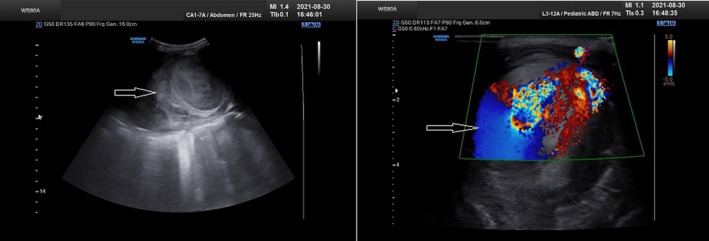
Ultrasound showed a large hematoma (arrow) posterior to the rectus muscle in the left lower quadrant. Superficial to the hematoma, there was an oval shape structure with turbulent flow in the Doppler study (to‐and‐fro pattern)

**FIGURE 3:**
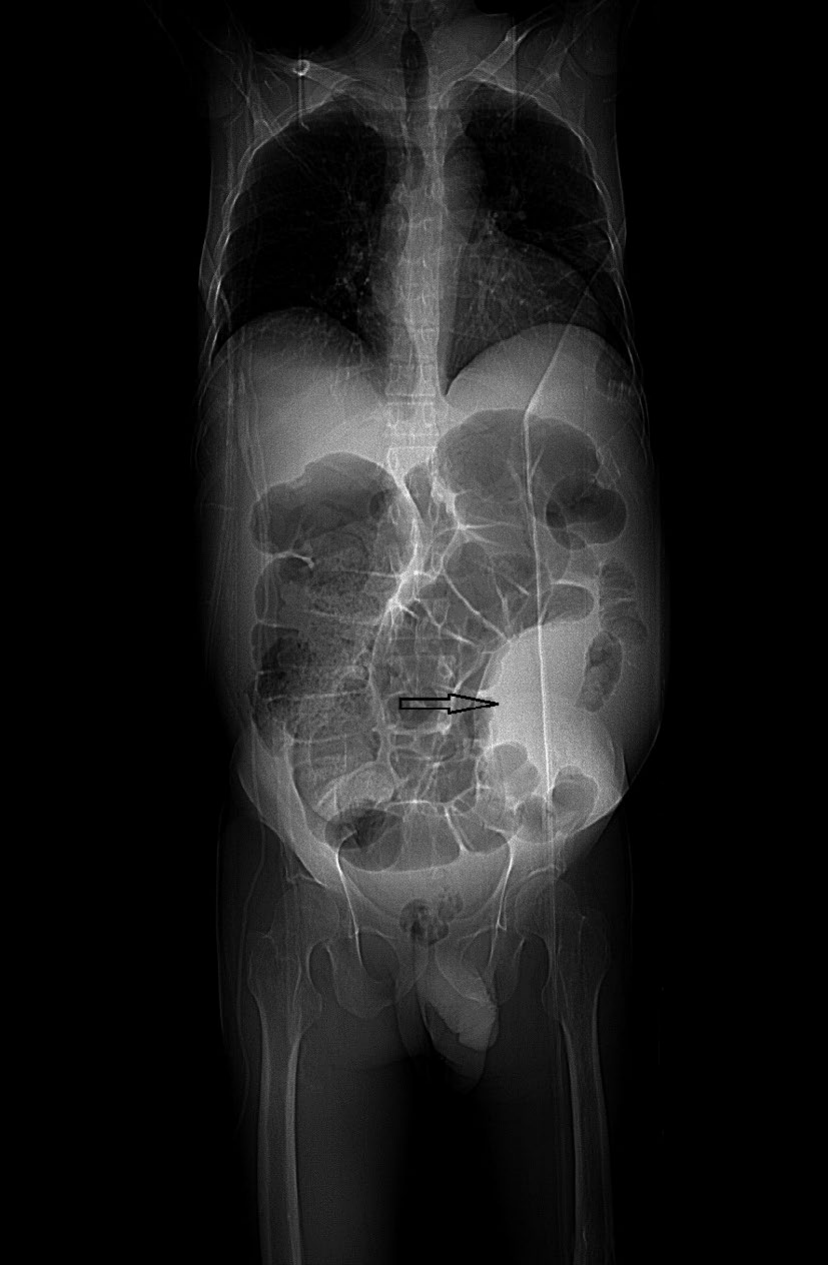
In the scout image taken from the patient, there was a soft tissue mass in the left lower quadrant (arrow)

**FIGURE 4:**
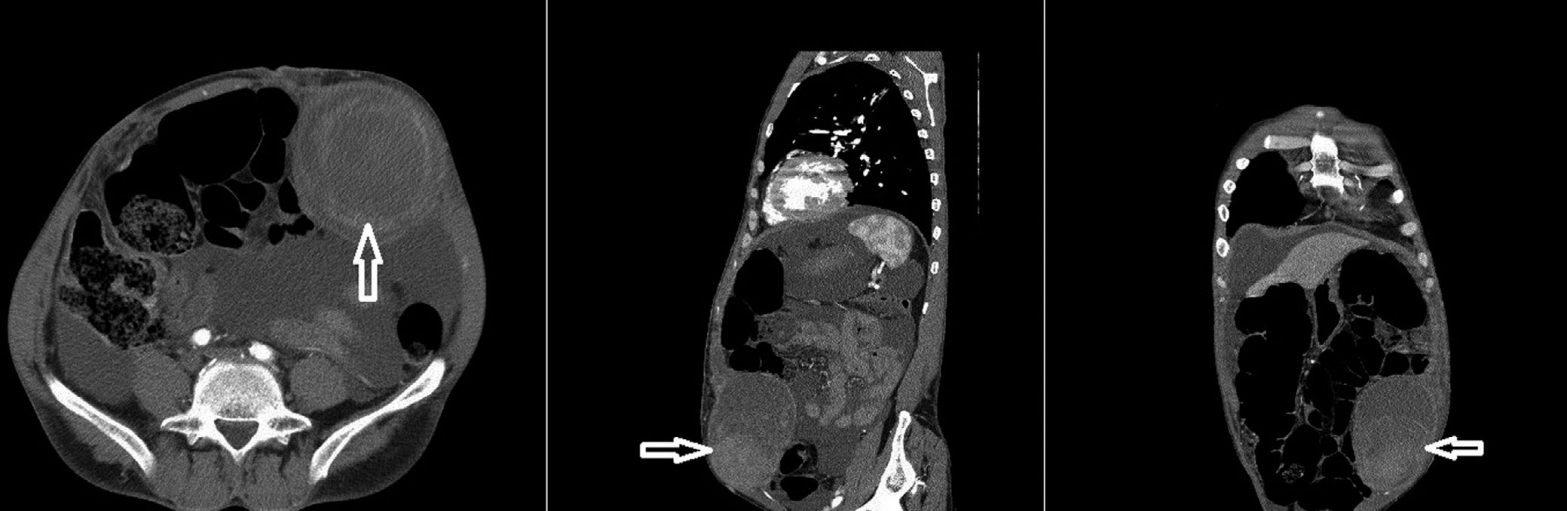
Contrast‐enhanced computed tomography (CT) section of the abdomen and pelvic demonstrated an oval circumscribed structure in the left rectus muscle with mixed attenuation. (arrows)

The patient was hemodynamically stable and was sent to the radiology department for an ultrasound examination. In the ultrasound performed for the patient, severe ascites and a large hematoma measured about 9.5 cm × 7.5 cm posterior to the rectus muscle in the left lower quadrant region were diagnosed. Superficial to the hematoma, an oval shape structure measured approximately 5.5 cm × 4.5 cm with the turbulent flow was detected. IEA pseudoaneurysm was confirmed in the Doppler study (Figure 2). The patient was transferred to a better‐equipped center where a computed tomography (CT) scan of the abdomen and pelvic with intravenous (IV) contrast was performed immediately. CT scan demonstrated a heterogeneous high‐density structure in the left rectus muscle (Figures 3 and 4).

The fascia was opened via a miniature hockey stick incision in the operation room, and the IEA was ligated. Then, an incision was made on the pseudoaneurysm, and content was evacuated. Also, the orifice of IEA was ligated from the inside of the pseudoaneurysm.

The pseudoaneurysm was diagnosed by Doppler ultrasound and treated by surgical intervention regarding the patient's underlying comorbidity. IEA false aneurysm must be included in the differential diagnosis during the investigation of the cause of any swelling after paracentesis.^4^

## 3 DISCUSSION

In general, the IEA arises from the external iliac artery immediately above the inguinal ligament. After leaving the external iliac artery, the IEA curves forward in the subperitoneal tissue and then ascends obliquely along the medial margin of the abdominal inguinal ring and behind the entrance of the spermatic cord.^2^ The anatomical position of the IEA subjects it to risk of injury during abdominal procedures close to the artery, such as laparoscopic trocar insertion, insertion of intra‐abdominal drains, Tenckhoff^®^ catheter (peritoneal dialysis catheter), and paracentesis.^5^ Paracentesis of the abdominal cavity is carried out to analyze ascitic fluid for diagnostic and therapeutic purposes. In recent years, the modern ultrasound‐guided method has favored the landmark‐based approach as the latter carries a higher risk of complications.^6^ Paracentesis is a safe procedure; however, possible complications include the following: Persistent leakage of asities fluid at the needle insertion site, abdominal wall hematoma, infection, perforation of surrounding vessels or viscera (extremely rare), Hypotension after large‐volume fluid removal (more than 5–6 L) and subcutaneous edema due to leakage of ascetic fluid.^7^, ^8^, ^9^

A pseudoaneurysm is a collection of blood formed outside a vessel, within the surrounding soft tissues. They are formed due to a connection or channel between blood collection and the damaged blood vessel. It differs from a true aneurysm where blood collection is contained within all three layers of the artery itself.^1^ IEA pseudoaneurysm is challenging to diagnose clinically. It usually presents as a diffuse, tender mass that is non‐pulsatile, and no bruit may be auscultated on it.^10^ So, it can be similar to simple hematomas, especially those occurring after procedures, including paracentesis that can cause hematoma as a complication.^4^ Any procedures penetrating the abdominal wall can therefore risk injury to the IEA. Although IEA pseudoaneurysms remain a rare event, the incidence rate appears to increase gradually, with 69% of the 32 known cases from the last 40 years presenting since 2000.^11^ When IEAI is suspected, CT angiography should be performed as the test of choice. However, negative findings on CT angiography should not deter catheter angiography when there is clinical evidence of ongoing bleeding. Alternative strategies for patients with increased creatinine levels who have severe contrast agent allergies include Doppler US or tagged red blood cell scanning.^12^, ^13^ Treatment choices for this rare entity include surgical excision and ligation, percutaneous procedures, and conservative management.^10^ In the present case, surgical excision and ligation were performed for the patient. General recommendations to prevent complications after paracentesis, such as pseudoaneurysm, include the following: avoiding surgical scars; avoiding the upper quadrants because of possible hepatosplenomegaly; staying away from the rectus muscles because of the superior and inferior epigastric vessels; avoiding large visible collateral venous channels; correcting coagulopathy with fresh‐frozen plasma; and using a small‐gauge needle. Ideally, the lateral lower quadrants and midline are best suited for paracentesis.^14^

## 4 CONCLUSION

In conclusion, the EIA pseudoaneurysm is a rare condition that occurs as a rare complication of paracentesis. We presented a case of EIA pseudoaneurysm which resulted in an iatrogenic setting. This rare condition reminds us to attend to the technical standards to prevent complications. The safe approach for paracentesis is lateral lower quadrants and midline. The treatment according to the size, location of the aneurysm, and patient's general condition can be varied. Our case was undergoing IEA ligation through a miniature hockey stick incision.

## CONFLICT OF INTEREST

We declare that we have no conflict of interest.

## AUTHOR CONTRIBUTIONS

Dr. Behnam Kian involved in conceptualization, writing the initial draft, supervision, and validation. Dr. Arash Teimouri involved in data curation, writing the initial draft, and revision.

## ETHICAL APPROVAL

This study was approved by the ethical committee of Shiraz University of Medical Sciences.

## CONSENT

We have obtained the patient's consent for publication before inclusion in this study.

## Data Availability

Data sharing is not applicable to this article as no new data were created or analyzed in this study.
